# Supplementary material for: Engineering artificial photosynthetic life-forms through endosymbiosis
Source: Nat Commun. 2022 Apr 26;13:2254. doi: 10.1038/s41467-022-29961-7 (PMC9042829; doi:10.1038/s41467-022-29961-7)
Supplement: Supplementary file 1 — Supplementary Information [file 41467_2022_29961_MOESM1_ESM.pdf]

Engineering artificial photosynthetic life-forms through endosymbiosis

Cournoyer *et al.*

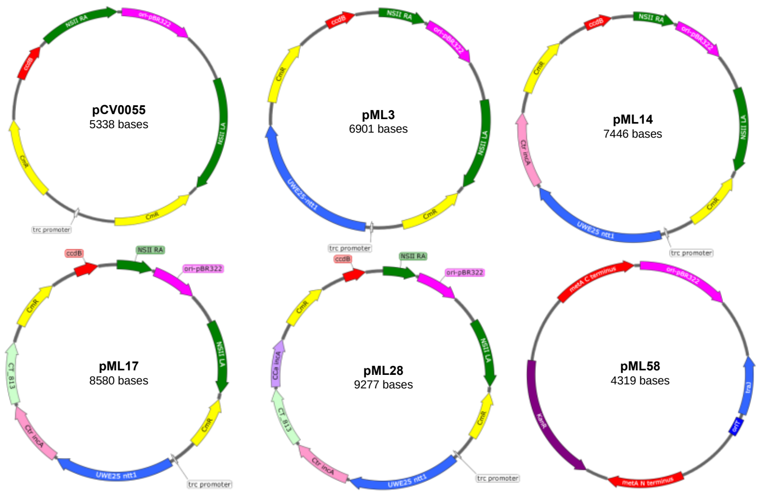

Supplementary Fig. 1. Plasmids used in this study. UWE25 ntt1 – ADP/ATP translocase; Ctr-incA, Cca_incA and CT_813 are genes encoding SNARE-like proteins from *C. trachomatis*, *C. caviae and C. trachomatis*, respectively; CmR – chloramphenicol acetyltransferase; KanR – aminoglycoside-3'-phosphotransferase; trc is a constitutive promoter; NSII, metA N-terminus and metA C terminus are all homologous recombination sites at the 5’-end and 3’-end of the *metA* gene in the Syn7942 genome.

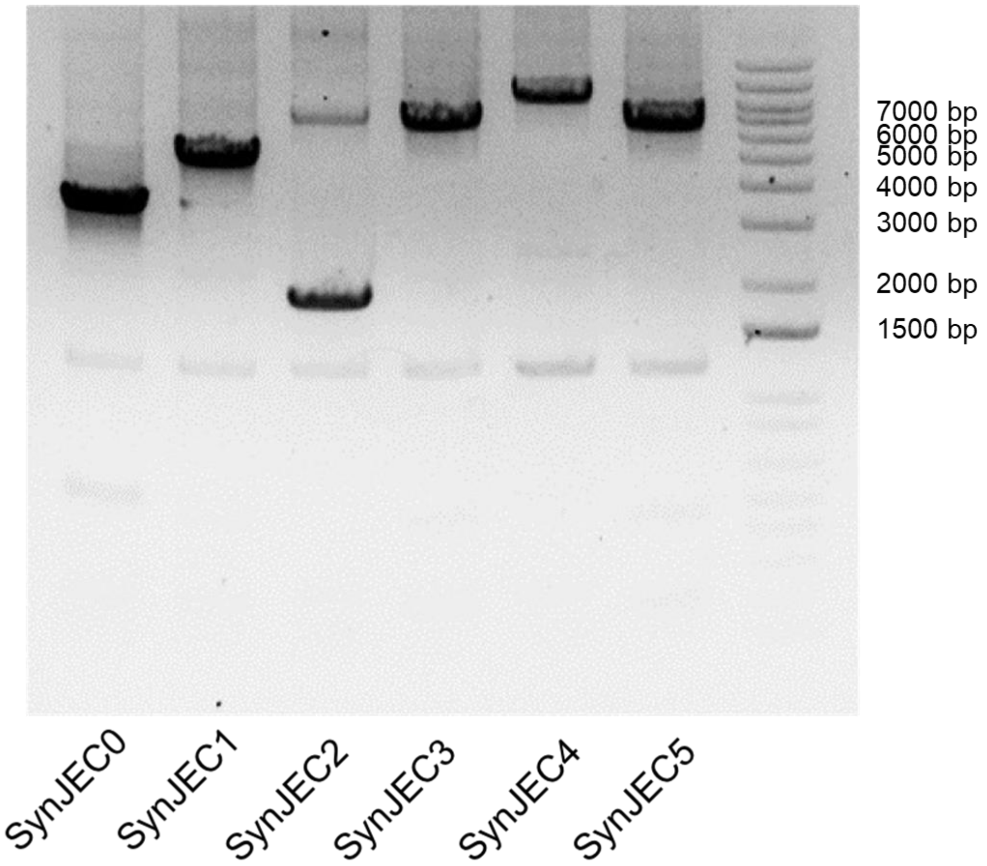

Supplementary Fig. 2. PCR amplification of the *NSII* locus of Syn7942 to confirm recombination: Recombination was verified by DNA sequencing analysis of amplified and gel purified DNA fragment. The experiment was repeated twice independently with similar results. Source data are provided as a Source Data file

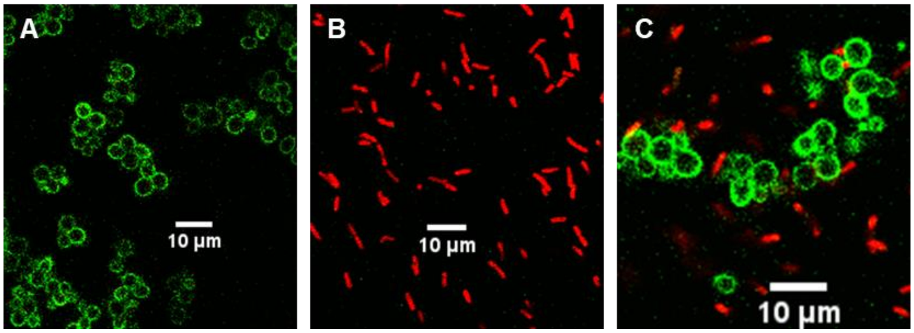

Supplementary Fig. 3. Confocal image for controls. (A) yeast only (pseudo-color: green), **(B)** cyanobacteria only (pseudo-color: red), **(C)** yeast/cyanobacteria mix. The experiment was repeated twice independently with similar results.

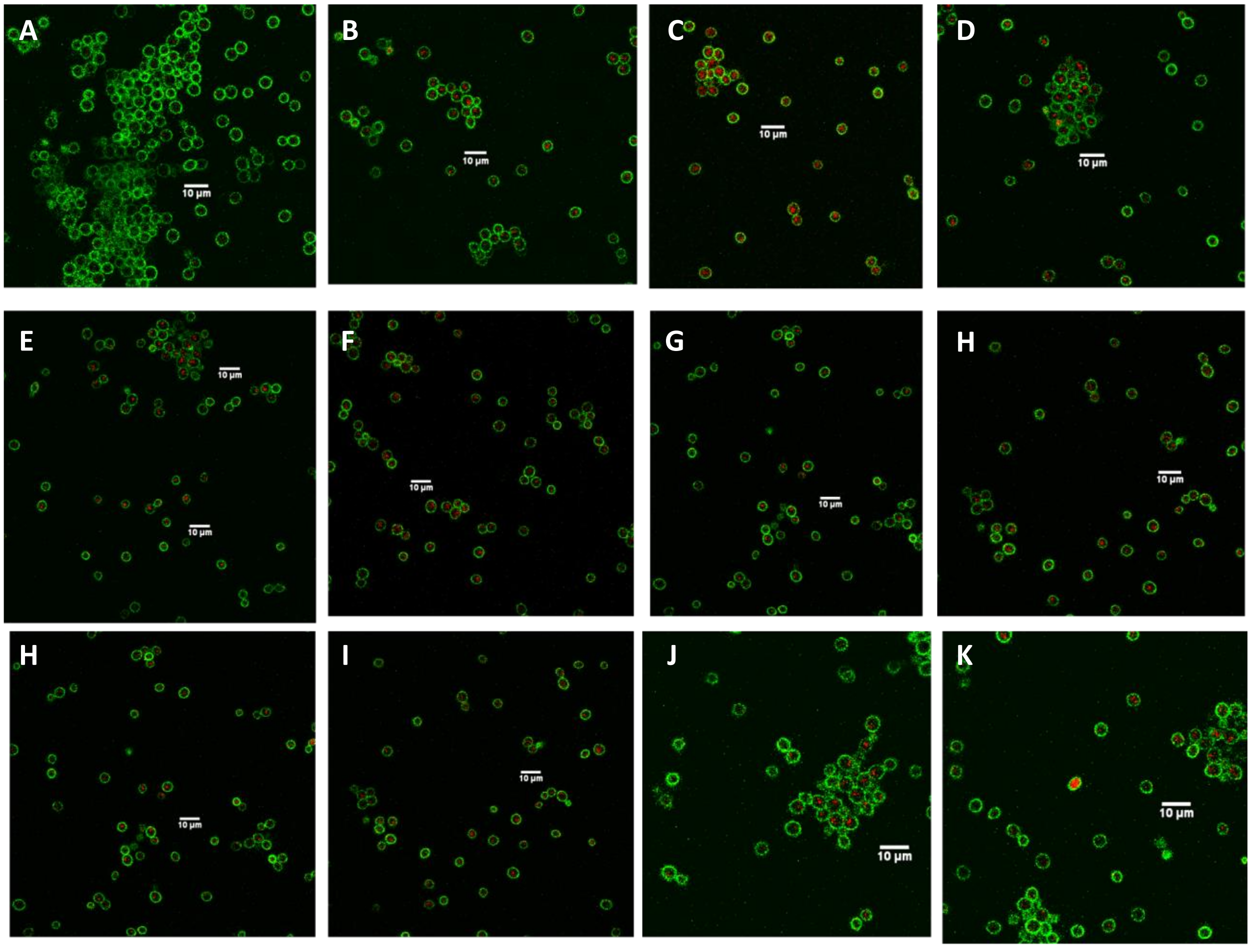

Supplementary Fig. 4. Tracking the yeast/cyanobacteria chimeras during various stages of selection using fluorescence confocal microscopy. (A) Control yeast cells **(B)-(K)** Images of early stages (5-10 doublings) to late stages Yeast/SynJEC3 (15-20 doublings) chimeras propagated for multiple rounds of selection imaged by fluorescence confocal microscopy. The yeast cell wall was stained with Con A-FITC (pseudo-color: green, Ex. = 488 nm; Em. = 510/20) and presence of cyanobacteria was monitored by cyanobacterial fluorescence (pseudo-color: red, Ex. = 561 nm; Em. = 650/20). The experiments were repeated twice independently with similar results.

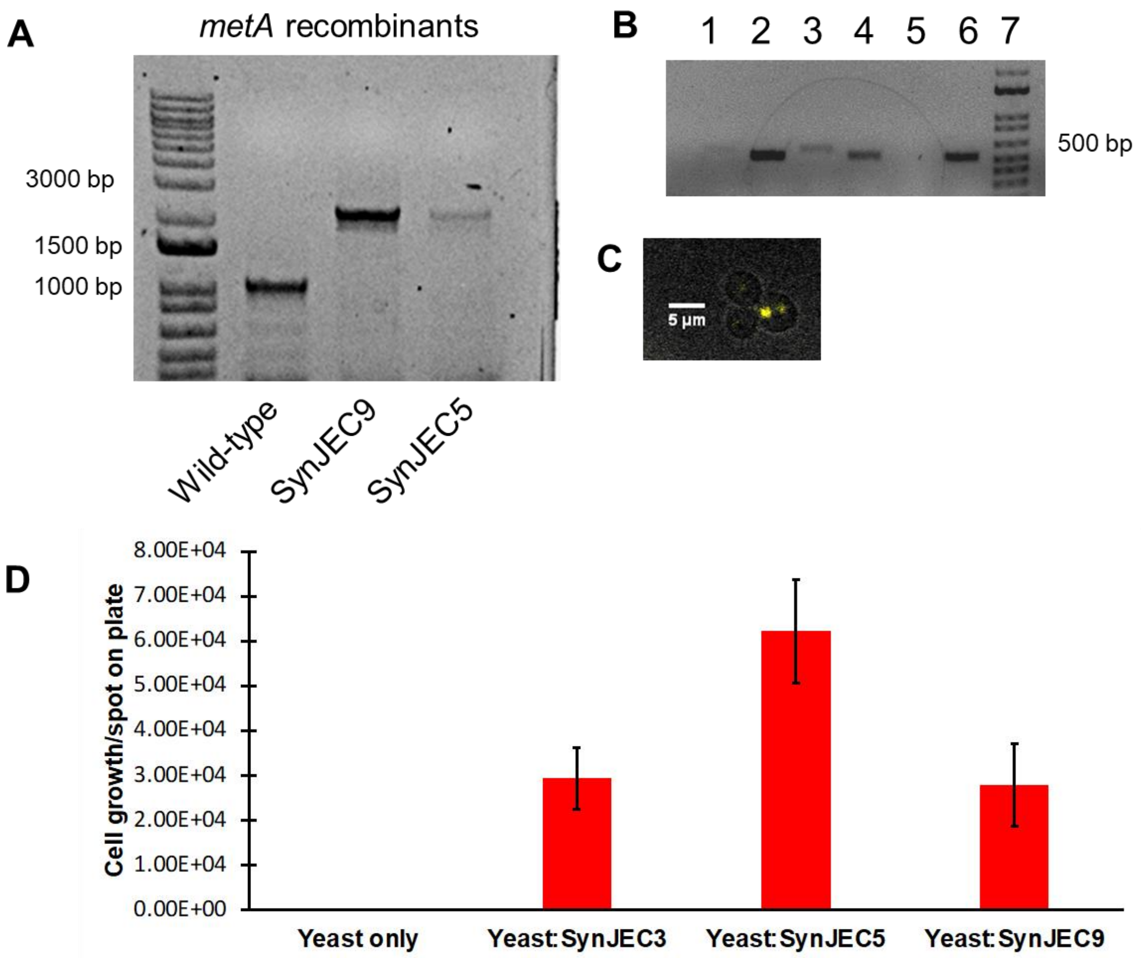

Supplementary Fig. 5. Yeast-SynJEC chimeras containing endosymbionts with methionine auxotrophs. (A) PCR amplification of the metA locus to confirm *metA* deletion. The experiment was repeated twice independently with similar results. **(B)** Analysis of total genomic DNA from Yeast-SynJEC chimeras. Lane 1: PCR amplification of *CAT* from yeast:SynJEC9 chimeras; Lane 2: PCR amplification of *MATa* from yeast:SynJEC9 chimeras; Lane 3: PCR amplification of *CAT* from yeast:SynJEC5 chimeras; Lane 4: PCR amplification of *MATa* from yeast:SynJEC5 chimeras; Lane 5: PCR amplification of *CAT* from yeast only; Lane 2: PCR amplification of *MATa* from yeast only; Lane 7: Standards. The experiment was repeated twice independently with similar results. **(C)** TIRF microscopy image of yeast:SynJEC5 chimeras (pseudo-color: yellow corresponds to pTIRF/excitation with 561 nm laser with open shutter; black and white image corresponds to pTIRF with closed laser shutter. The experiment was repeated three times independently with similar results. **(D)** Growth trends (at round IV of repropagation) of *S. cerevisiae cox2-60* (yeast only strains), *S. cerevisiae cox2-60-* SynJEC3 (yeast-SynJEC3), *S. cerevisiae cox2-60- SynJEC5* (yeast-SynJEC3) and *S. cerevisiae cox2-60- SynJEC9* (yeast-SynJEC4) chimeras on Selection Medium III. Cells (3.00×10^3) were spotted on Selection Medium III and the final number of cells/spot on plate were determined after 48 h of growth (n = 3 measurements per sample; error bars represent standard error of the mean). Source data are provided as a Source Data file.

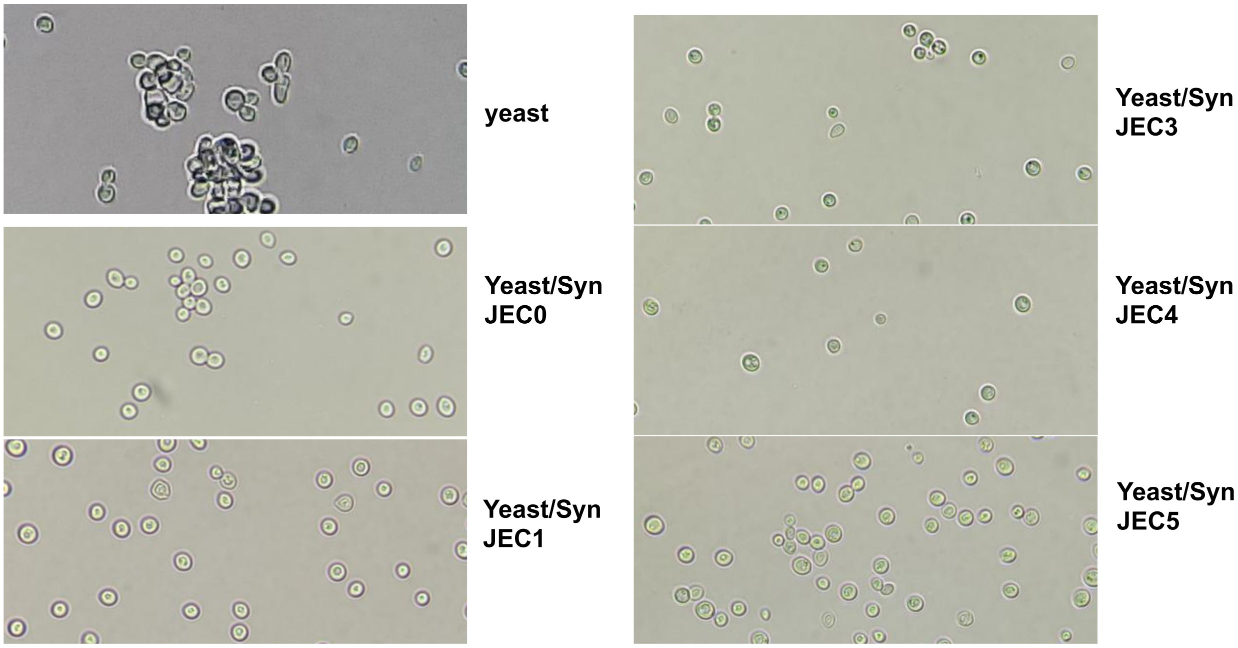

Supplementary Fig. 6. Representative microscopic images of the of the yeast/cyanobacteria chimera that were used for the isolation of the total genomic DNA and PCR analysis. Using a combination of these images and TIRF images, we do not detect the presence of extracellular cyanobacterial cells under these conditions. The experiment was repeated six times independently with similar results.

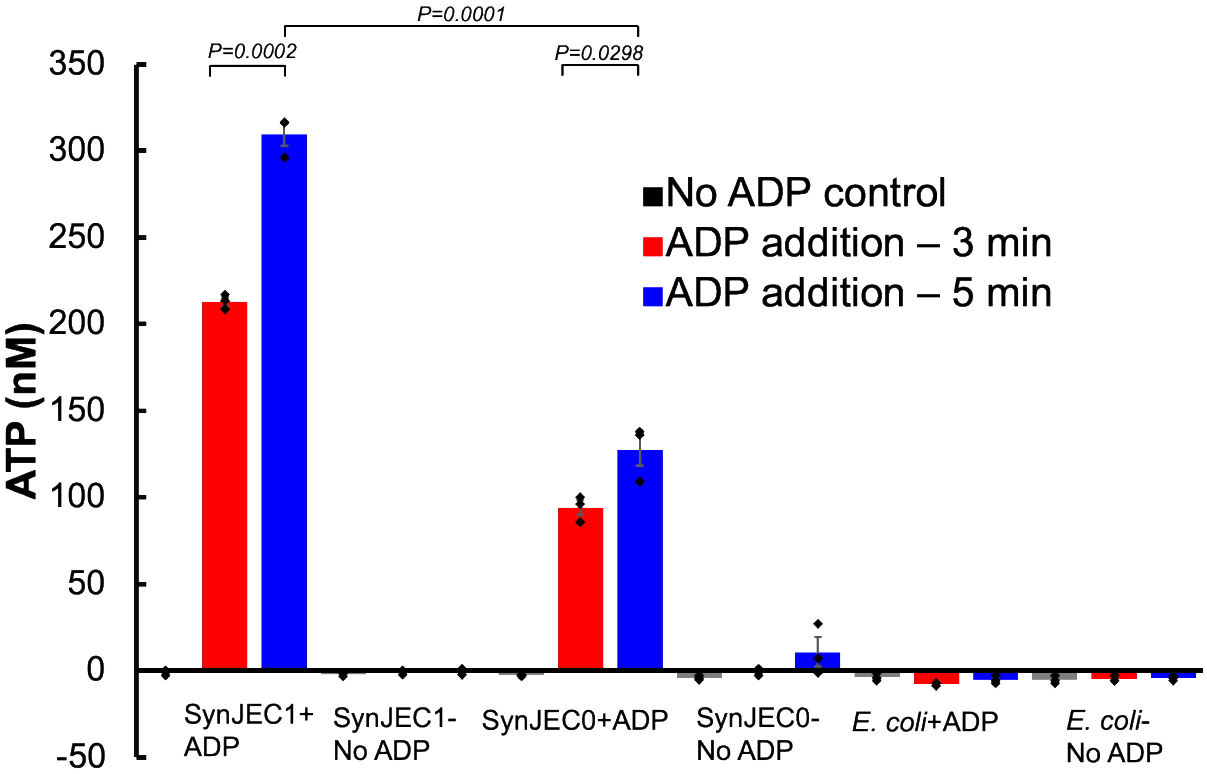

Supplementary Fig. 7. ADP/ATP translocase assays: Comparison of SynJEC strains to *E. coli* DH10B cells. Release of ATP by SynJEC1 cells expressing the UWE25 ADP/ATP translocase in the presence of 80 µM ADP in comparison to SynJEC0 cells. ATP was released when SynJEC1 (expressing the ATP/ADP translocase) and SynJEC0 cells were challenged with extracellular ADP (80 µM), but not with a blank solution lacking ADP. ATP was not released when *E. coli* DH10B were challenged with extracellular ADP (80 µM). N=3 biologically independent experiments. Data are presented as mean values +/- SEM. Two-sided t-tests were used to compare means without adjustments (95% CI, Cohen's d=10.6, DF=4, P=0.0002; 95% CI, Cohen's d=13.0, DF=4, P=0.0001; 95% CI, Cohen's d=2.7, DF=4, P=0.0002). Source data are provided as a Source Data file.

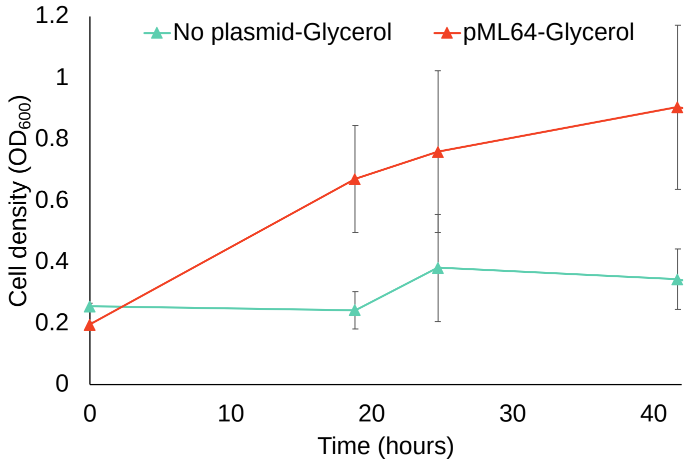

Supplementary Fig. 8. Growth rate of *S. cerevisiae cox2-60* under selection and non-selection conditions: Red trace - *S. cerevisiae cox2-60-pML64* growth rate under selection conditions containing glycerol as the carbon source. Cyan trace - *S. cerevisiae cox2-60* growth rate under selection conditions containing glycerol as the carbon source. N=24 biological replicates. The experiment was repeated twice independently with similar results. Data are presented as mean values +/- standard deviation. Source data are provided as a Source Data file

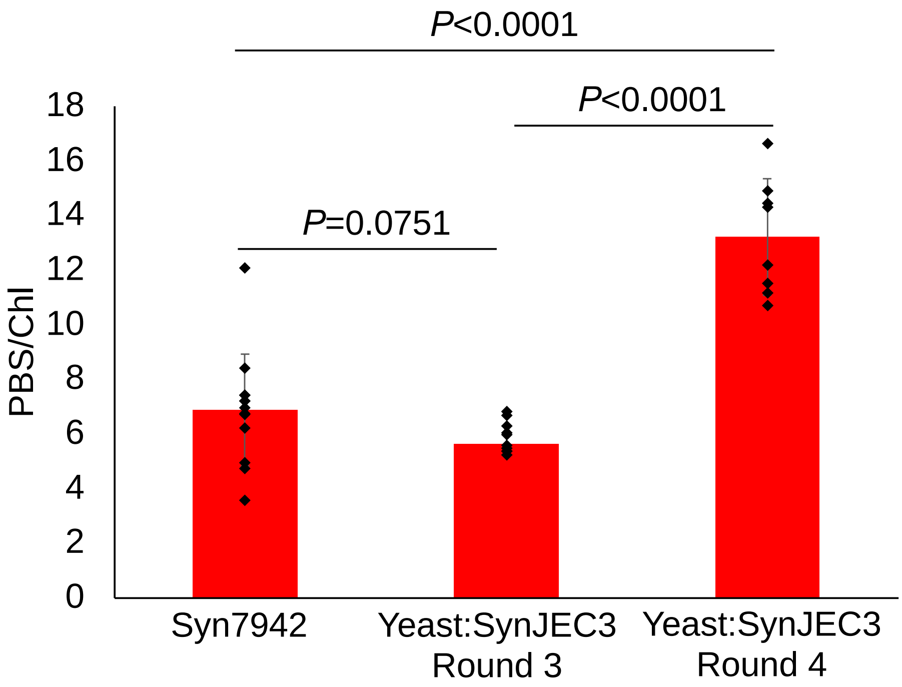

Supplementary Fig. 9. pTIRF microscopy to determine the relative changes in phycobilisomes and chlorophyll *a*. The relative levels of phycobilisomes (PBS, Ex. 561 nm) and chlorophyll (Chl, Ex. 405 nm) a as plotted as an internal ratio (PBS/Chl) with rounds of repropagation under selection conditions. N=13 (Syn7942), 10 (Yeast:SynJEC3 Round 3), 8 (Yeast:SynJEC3 Round 4) measurements of different ROIs taken from a single captured pTIRF image. The experiment was repeated twice independently with similar results. Data are presented as mean values +/- standard deviation. Two-sided t-tests were used to compare means without adjustments (95% CI, Cohen's d=0.8, DF=21, P=0.0751; 95% CI, Cohen's d=3.0, DF=19, P<0.0001; 95% CI, Cohen's d=4.9, DF=16, P<0.0001). Source data are provided as a Source Data file.

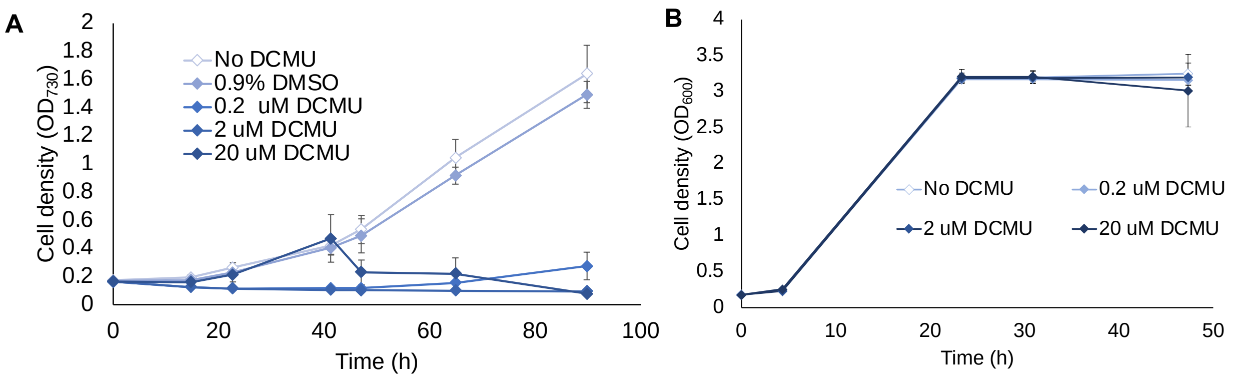

Supplementary Fig. 10. Effect of DCMU on the yeast and cyanobacteria growth. (A) Effect of DCMU on the growth of Syn7942. (B) Effect of DCMU on the growth of *S. cerevisiae cox2-60*. N=12 biological replicates. The experiment was repeated twice independently with similar results. Data are presented as mean values +/- standard deviation. Source data are provided as a Source Data file.

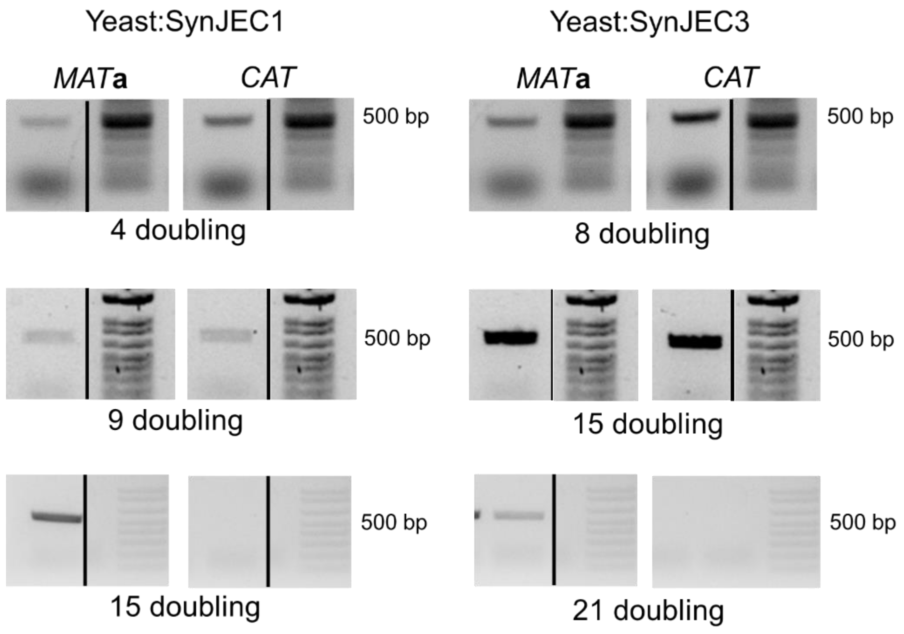

Supplementary Fig. 11. PCR analysis of total genomic DNA isolated from chimeras at various stages of growth. To characterize the presence of cyanobacterial endosymbionts within yeast cells, we isolated total genomic DNA from chimeras propagated under selection conditions for various rounds of growth. Presence of yeast genome is detected by amplification of yeast *MAT***a** gene and presence of SynJEC strains is detected by SynJEC chloramphenicol acetyltransferase (*CAT*) gene. After more than 20 rounds of doublings, we observe that the chimeras lose their viability possibly due to loss of cyanobacteria. The experiment was repeated three times independently with similar results. Source data are provided as a Source Data file

Supplementary Table 1. Genomic DNA fragments used in the construction of pML58.

Fragment name	Fragment sequence (5'->3')
metA-N term	GAAGAACAAGCCGCAGCTGGCGTCAGCCCTGACTTTGTGCGGCT<br>TTCGGTTGGCTTGGAGCATTTAGACGACATCTTGGCTGACCTTG<br>ACCAAGCCCTGCAGGCTTCGCAAGTCGCCTAGGTTGTTTCGTAC<br>TGGGCTTGCGATCGCCGACCCTCCCAGCGTTTTGCCATGCCGAT<br>CATCATTCCGCAAGATCTGCCTGCTCGTCGCATTCTGGATGCTG<br>AGTCCGTATTCACGCTGGGGGATGCGGATGCGCGGCGGCAGGA<br>TATCCGCGCTTTGCAAGTGGTTGTTTTGAACCTAATGCCGACCA<br>AGGTGACAACCGAAACGCAGATTGCTCGGGTACTCGCCAATAC<br>GCCGCTACAAGTAGAACTCACTTTAATTCACACGGCTAGCTATC<br>AACCAACCCACACCGACCCCGAGCATCTTCGTAACTTCTACAGC<br>ACCTTTGATCAAATTCGCGATCGCCAGTTTGATGGCTTGATTGT<br>GA
metA-C term	TGACGACTATTCTGGATTGGAGTCGTGAGGCGGTGCGCTCCAGT<br>TTGTTCATCTGCTGGGGAGCCCAAGCCGCGCTTCAGCATTTTCA<br>TGGCATTGAAAAGCAAACCTTGCCAGCCAAGCGCTTTGGCGTTT<br>TTTGGCATCATCTCCGCGATCGCAGTTCTCCCTTGGTACGCGGCC<br>ACGATGATGATTTTCTGGTGCCGGTCAGTCGCCATACGGAGGTA<br>ATTGCGGCTGAGGTATTGGCTCAATCACAGTTGCAAATTCTGGC<br>AGAAAGCTCAGAGGCTGGACTCCATCTCCTCTGGGATGCAGACC<br>AACATCGCACCTATCTGTTCAACCATCCGGAATACGATGCAGAC<br>ACCCTCGATCGCGAATATCGACGCGATCGCGAGAAAGGGTTGC<br>CGATTCAGTTACCTCTCAACTACTACCCTAATGATGACCCGAAT<br>CAAGTGCCGAGAGTGCGTTGGCGTAGCCATGCTCAACTGCTTTA<br>CACTAACTGGCTAAACTACGAGGTTTATCAACCACTGTCACGCT<br>AA

Supplementary Table 2. Oligonucleotides used for analysis of the total genomic DNA isolated from the yeast/cyanobacteria chimera.

Oligonucleotide name	Oligonucleotide sequence (5'->3')
AM965	AGTCACCATCAAGATCGTTTATGG
AM966	GCACGGAATATGGGACTACTTCG
AM967	ACTCCACTTCAAGTAAGAGTTTG
AM1224	CAAAATGGAGAGTTTGATCCTGGCTCAGG
AM1225	AAAGGAGGTGATCCAGCCACACCT
AM1351	TCAAGACGACTTGGTACTAGGACTCG
AM1456	ATGCCAATGACAACCCCTACGTTG
C2F	GCCTTGCCATTCATCACAGTAC
C9R	CCGCAAAGAACACTTTGAAGCC

Supplementary Table 3. Vector map links for maps listed in this paper.

Plasmid name	Benchling link
pCV0049	https://benchling.com/s/seq-P2Wv1DvB3F5HeufjL2IH
pCV0055	https://benchling.com/s/seq-QlPIEWddeBGaxRumfGBm
pML3	https://benchling.com/s/seq-XugIBKLDymnz5C6tVHm8
pML14	https://benchling.com/s/seq-OYZhEqEUAERMC4k36bqg
pML17	https://benchling.com/s/seq-BnSbm61OdiyAK1pvOXcg
pML28	https://benchling.com/s/seq-bl7pi8OvkcpczeVMVgNn
pML58	https://benchling.com/s/seq-lRLmm9gNLVt0jO3h4Smk
pML59	https://benchling.com/s/seq-gY35CjgwmBJEBfxfooAh?m=slm-j56wvCpY7Q3qazIi36ZL
pML60	https://benchling.com/s/seq-sT6JgJbNyzsiPXVxcVHy?m=slm-16xb853pom6UG8po4LPd
pML62	https://benchling.com/s/seq-CGCepat4VSOKikZ1vPHy
pML64	https://benchling.com/s/seq-P2Wv1DvB3F5HeufjL2IH

Supplementary Table 4. Oligonucleotides used for analysis of genomic DNA isolated from recombinant Syn7942 mutants.

Oligonucleotide name	Oligonucleotide sequence (5’->3’)
LL56	GAAGAACAAGCCGCAGC
LL57	TTAGCGTGACAGTGGTTGA
JC192	TCTGGATGCGGTGACTTGGCA

Supplementary Table 5. Comparison of total doublings detected for the key yeast/cyanobacteria chimera under optimal growth conditions in selection medium III.

Yeast/Cyanobacteria chimera	Total doublings detected
S. cerevisiae cox2-60	2
S. cerevisiae cox2-60-SynJEC0	11
S. cerevisiae cox2-60-SynJEC1	14
S. cerevisiae cox2-60-SynJEC3	22
