# Supplementary material for: Engineering artificial photosynthetic life-forms through endosymbiosis
Source: Nat Commun. 2022 Apr 26;13:2254. doi: 10.1038/s41467-022-29961-7 (PMC9042829; doi:10.1038/s41467-022-29961-7)
Supplement: Supplementary file 3 — Description of Additional Supplementary Files [file 41467_2022_29961_MOESM3_ESM.pdf]

Description of Additional Supplementary Files:

File Name: Supplementary Data 1
Description: Sequences of double stranded DNA (gblocks) commercially obtained from Integrated DNA Technologies (IDT)

File Name: Supplementary Data 2
Description: Sequences of single stranded DNA oligonucleotides commercially obtained from Integrated DNA Technologies (IDT)
