# Supplementary material for: Engineering artificial photosynthetic life-forms through endosymbiosis
Source: Nat Commun. 2022 Apr 26;13:2254. doi: 10.1038/s41467-022-29961-7 (PMC9042829; doi:10.1038/s41467-022-29961-7)
Supplement: Supplementary file 6 — Reporting Summary [file 41467_2022_29961_MOESM6_ESM.pdf]

nature research

Corresponding author(s): Angad P. Mehta

Last updated by author(s): 03302022

Reporting Summary

Nature Research wishes to improve the reproducibility of the work that we publish. This form provides structure for consistency and transparency in reporting. For further information on Nature Research policies, see our Editorial Policies and the Editorial Policy Checklist.

Statistics

For all statistical analyses, confirm that the following items are present in the figure legend, table legend, main text, or Methods section.

n/a	Confirmed	
☐	☒	The exact sample size (*n*) for each experimental group/condition, given as a discrete number and unit of measurement
☐	☒	A statement on whether measurements were taken from distinct samples or whether the same sample was measured repeatedly
☒	☐	The statistical test(s) used AND whether they are one- or two-sided<br>*Only common tests should be described solely by name; describe more complex techniques in the Methods section.*
☒	☐	A description of all covariates tested
☒	☐	A description of any assumptions or corrections, such as tests of normality and adjustment for multiple comparisons
☐	☒	A full description of the statistical parameters including central tendency (e.g. means) or other basic estimates (e.g. regression coefficient) AND variation (e.g. standard deviation) or associated estimates of uncertainty (e.g. confidence intervals)
☐	☒	For null hypothesis testing, the test statistic (e.g. *F*, *t*, *r*) with confidence intervals, effect sizes, degrees of freedom and *P* value noted<br>*Give P values as exact values whenever suitable.*
☒	☐	For Bayesian analysis, information on the choice of priors and Markov chain Monte Carlo settings
☒	☐	For hierarchical and complex designs, identification of the appropriate level for tests and full reporting of outcomes
☒	☐	Estimates of effect sizes (e.g. Cohen's *d*, Pearson's *r*), indicating how they were calculated

Our web collection on statistics for biologists contains articles on many of the points above.

Software and code

Policy information about availability of computer code

Data collection	Gen5 v. 3.11 was used for luminescence and OD measurements. TIRFm for TIRF. Leica Application Suite (LASX) for confocal microscopy. IDT codon optimization (https://www.idtdna.com/CodonOpt).
Data analysis	ImageJ 1.53c. for microscopy images. All other data were processed in Microsoft Excel.

For manuscripts utilizing custom algorithms or software that are central to the research but not yet described in published literature, software must be made available to editors and reviewers. We strongly encourage code deposition in a community repository (e.g. GitHub). See the Nature Research guidelines for submitting code & software for further information.

Data

Policy information about availability of data

All manuscripts must include a data availability statement. This statement should provide the following information, where applicable:

- Accession codes, unique identifiers, or web links for publicly available datasets
- A list of figures that have associated raw data
- A description of any restrictions on data availability

Plasmid maps and links are provided with the paper. Source data are provided with the paper

Field-specific reporting

Please select the one below that is the best fit for your research. If you are not sure, read the appropriate sections before making your selection.

☒ Life sciences ☐ Behavioural & social sciences ☐ Ecological, evolutionary & environmental sciences

For a reference copy of the document with all sections, see nature.com/documents/nr-reporting-summary-flat.pdf

Life sciences study design

All studies must disclose on these points even when the disclosure is negative.

Sample size	No statistical methods were used to determine the sample size. Sample size of 12-24 was used for growth assays of microorganisms in order to ensure reproducibility and minimize error intrinsic to 96-well plate assays. Biochemical experiments were performed with sample size of N=3 biological replicates to ensure reproducibility while minimizing cost. In these instances, biochemical assays were repeated independently by different researchers. All sample size were determined from preliminary test experiments.
Data exclusions	No data were excluded from analysis
Replication	All experiments were reproduced at least two times
Randomization	No randomization was applied to this study. No animals or human subjects were involved. Randomization was not necessary because experiments provided quantitative results. Experiments provided consistent quantitative results and were executed by at least three investigators. Covariates/uncontrollable conditions of the experiment did not affect the results, because controls were always included in assays to avoid bias. Materials used for biochemical assays were prepared fresh (e.g. reagents, cells, etc.) were prepared freshly each time the experiments were performed to ensure contamination did not effect the materials.
Blinding	Blinding was not performed because the data we analyzed are quantitative and included controls. This is a proof-of concept microbial synthetic biology study. Source data are provided with the paper showing loading controls. Source materials are available from the authors. engineering study

Reporting for specific materials, systems and methods

We require information from authors about some types of materials, experimental systems and methods used in many studies. Here, indicate whether each material, system or method listed is relevant to your study. If you are not sure if a list item applies to your research, read the appropriate section before selecting a response.

Materials & experimental systems

n/a	Involved in the study	
☒	☐	Antibodies
☐	☒	Eukaryotic cell lines
☒	☐	Palaeontology and archaeology
☒	☐	Animals and other organisms
☒	☐	Human research participants
☒	☐	Clinical data
☒	☐	Dual use research of concern

Methods

n/a	Involved in the study	
☒	☐	ChIP-seq
☒	☐	Flow cytometry
☒	☐	MRI-based neuroimaging

Eukaryotic cell lines

Policy information about cell lines

Cell line source(s)	Saccharomyces cerevisiae cox2-60 was donated by the Schultz lab (Scripps Research)
Authentication	The cell lines were not authenticated.
Mycoplasma contamination	The cell lines were not tested for Mycoplasma contamination
Commonly misidentified lines (See ICLAC register)	No commonly misidentified cell lines were used in this study.
